# COVID-19 Vaccination Preferences Among Non-Chinese Migrants in Hong Kong: Discrete Choice Experiment

**DOI:** 10.2196/40587

**Published:** 2023-03-27

**Authors:** Saba Asim, Kailu Wang, Elena Nichini, Faustina Fu Yip, Liling Zhu, Hin Chung Eddy Fung, Yan Zeng, Zhilan Fang, Annie Wai-Ling Cheung, Eliza Lai-yi Wong, Dong Dong, Eng-Kiong Yeoh

**Affiliations:** 1 The Jockey Club School of Public Health and Primary Care The Chinese University of Hong Kong Shatin Hong Kong; 2 Centre for Health Systems and Policy Research The Jockey Club School of Public Health and Primary Care The Chinese University of Hong Kong Shatin Hong Kong; 3 Faculty of Law The Chinese University of Hong Kong Shatin Hong Kong

**Keywords:** COVID-19 vaccination, migrants, discrete choice experiment, ethnic minorities, vaccine attributes, Hong Kong, COVID-19

## Abstract

**Background:**

Studies have shown increasing COVID-19 vaccination hesitancy among migrant populations in certain settings compared to the general population. Hong Kong has a growing migrant population with diverse ethnic backgrounds. Apart from individual-level factors, little is known about the migrants’ preference related to COVID-19 vaccines.

**Objective:**

This study aims to investigate which COVID-19 vaccine–related attributes combined with individual factors may lead to vaccine acceptance or refusal among the migrant population in Hong Kong.

**Methods:**

An online discrete choice experiment (DCE) was conducted among adults, including Chinese people, non-Chinese Asian migrants (South, Southeast and Northeast Asians), and non-Asian migrants (Europeans, Americans, and Africans) in Hong Kong from February 26 to April 26, 2021. The participants were recruited using quota sampling and sent a link to a web survey. The vaccination attributes included in 8 choice sets in each of the 4 blocks were vaccine brand, safety and efficacy, vaccine uptake by people around, professionals’ recommendation, vaccination venue, and quarantine exemption for vaccinated travelers. A nested logistic model (NLM) and a latent-class logit (LCL) model were used for statistical analysis.

**Results:**

A total of 208 (response rate 62.1%) migrant participants were included. Among the migrants, those with longer local residential years (n=31, 27.7%, for ≥10 years, n=7, 20.6%, for 7-9 years, n=2, 6.7%, for 4-6 years, and n=3, 9.7%, for ≤3 years; *P*=.03), lower education level (n=28, 28.3%, vs n=15, 13.9%, *P*=.01), and lower income (n=33, 25.2%, vs n=10, 13.2%, *P*=.04) were more likely to refuse COVID-19 vaccination irrespective of vaccination attributes. The BioNTech vaccine compared with Sinovac (adjusted odds ratio [AOR]=1.75, 95% CI 1.14-2.68), vaccine with 90% (AOR=1.44, 95% CI 1.09-1.91) and 70% efficacy (AOR=1.21, 95% CI 1.03-1.44) compared with 50% efficacy, vaccine with fewer serious adverse events (1/100,000 compared with 1/10,000; AOR=1.12, 95% CI 1.00-1.24), and quarantine exemption for cross-border travelers (AOR=1.14, 95% CI 1.01-1.30) were the vaccine attributes that could increase the likelihood of vaccination among migrants. For individual-level factors, full-time homemakers (AOR=0.44, 95% CI 0.29-0.66), those with chronic conditions (AOR=0.61, 95% CI 0.41-0.91) and more children, and those who frequently received vaccine-related information from the workplace (AOR=0.42, 95% CI 0.31-0.57) were found to be reluctant to accept the vaccine. Those with a higher income (AOR=1.79, 95% CI 1.26-2.52), those knowing anyone infected with COVID-19 (AOR=1.73, 95% CI 1.25-2.38), those having greater perceived susceptibility of COVID-19 infection (AOR=3.42, 95% CI 2.52-4.64), those who received the influenza vaccine (AOR=2.15, 95% CI 1.45-3.19), and those who frequently received information from social media (AOR=1.52, 95% CI 1.12-2.05) were more likely to accept the vaccine.

**Conclusions:**

This study implies that migrants have COVID-19 vaccination preference heterogeneity and that more targeted and tailored approaches are needed to promote vaccine acceptance for different subgroups of the migrant population in Hong Kong. Vaccination promotion strategies are needed for low-education and low-income migrant groups, migrants with chronic diseases, the working migrant population, homemakers, and parents.

## Introduction

COVID-19 vaccination has been implemented worldwide as a crucial public health tool to reduce morbidity and mortality and eventually put an end to social distancing measures and the pandemic as a whole [1]. As of December 29, 2021, about 8.68 billion vaccine doses had been administered worldwide [2], first targeting priority groups and gradually extending vaccination to the general population.

For this campaign to be successful, public acceptance and the willingness to undergo vaccination are needed across the whole population [1,3]; yet research indicates that migrant populations in certain settings are particularly hesitant toward COVID-19 vaccines [4,5]. However, little is known about the migrants’ preference related to COVID-19 vaccines [6]; this undermines health equity, since as vulnerable populations, migrants are at a higher risk of COVID-19 infection, hospitalization, and mortality compared to the general population [7].

Previous studies have mainly focused on individual-level factors. They have reported that migrants’ socioeconomic conditions and gender have an impact on COVID-19 vaccine acceptance [3,8,9]. Ethnicity-related factors can influence vaccine hesitancy as well [4,9-11]. Moreover, the literature on migrant health suggests that migration history can differently affect access to primary medicine and vaccination uptake [12].

Finally, low vaccine coverage among migrants may be due to limited knowledge of the overall health care system [4]; in addition, during the pandemic, barriers within health care systems usually identified as possible factors affecting migrants’ lower access to preventive care and vaccinations have increased [5,9]. Trust in the health care system or in vaccines may affect vaccine uptake as well [4,9,11,13].

Hong Kong is an international city with a growing migrant and ethnic population, which reached 8% of the overall population in 2016, representing a sharp increase of about 70% compared to 2006 [14]. Despite its facade of an international and inclusive city, Hong Kong holds a hegemonic Chinese culture where exclusion is widespread [15]; this may affect migrant population access and attitudes toward vaccines as well.

Although research has focused on individual variables, little is known about how vaccine-related attributes may influence migrants’ attitudes toward COVID-19 vaccination. Similar to previous research conducted on the general population [16,17], this study aims to investigate COVID-19 vaccine preferences among the migrant population by identifying which individual factors combined with vaccine-related attributes may lead to vaccine acceptance or refusal. Although most of the literature focuses on the general population, this analysis will provide a more nuanced understanding of migrants’ overall attitudes toward vaccines and guide public health efforts to promote COVID-19 vaccination among more diverse groups.

## Methods

### Study Sample and Data Collection

An online discrete choice experiment (DCE) was conducted among all adults, including Chinese people and non-Chinese migrants in Hong Kong, China, from February 26 to April 26, 2021, the first 2 months after the commencement of the citywide COVID-19 vaccination program of 2 major vaccines. Chinese or non-Chinese residents of Hong Kong aged 18 years or above were eligible to participate in the survey, while those who had a history of being diagnosed with COVID-19 or had received any COVID-19 vaccine were excluded. The participants were recruited using quota sampling for people with different ethnicities according to Hong Kong census data [18], including Chinese, non-Chinese Asians (South Asians, Southeast Asians and Northeast Asians), and non-Asians (Europeans, Americans, and Africans). A web link to the survey was sent to potential participants through the network established in a previous survey, through local nongovernmental organizations that provide social services to socially disadvantaged individuals and migrants, and through persons familiar with the migrant communities. The distribution of ethnicities in the sample was monitored online during the survey to adjust the sampling strategies for the remaining participants, and ceiling limits (quotas) were set for the number of participants with different ethnicities based on the ethnical distribution in the population. The survey was originally designed in English and then forward- and back-translated to other languages and thus was also available in Bahasa Indonesia, Nepali, Urdu, Thai, and traditional Chinese.

### Experimental Design

A DCE requires participants to make choices from a series of choice sets described by a number of attributes. The attributes describing the COVID-19 vaccination plans included vaccine brand, efficacy, safety, uptake of vaccine by people around, recommendations from professionals, venue for vaccination, and exemption of quarantine for vaccinated travelers (Table 1), which were generated from prior individual interviews with 45 Hong Kong residents with diverse demographical characteristics and health conditions [19]. In this qualitative study, the confidence in and concerns about the benefits and side effects of the vaccines, vaccine origins and brands, recommendations from health care professionals, social influences of family members and friends, expectations of ease of travel restrictions, and logistics arrangement of vaccination (eg, locations of receiving the vaccines) were frequently reported as factors influencing the participants’ willingness to accept the vaccines [19]. The levels of the attributes were determined based on the existing studies on vaccine effectiveness and safety [20-23] and the vaccination practice in Hong Kong [24]. For vaccine brands, all 3 (Sinovac, BioNtech, and AstraZeneca) were planned to be used for vaccination in Hong Kong at the time of the study, although only the former 2 of them were actually launched for the public eventually.

The full factorial design of the 7 attributes involved 648 combinations and 209,628 pairwise choice sets, so it was impossible to adopt the full factorial design in the DCE. The choice sets were designed using the D-optimality algorithm, and a total of 32 choice sets were generated. To further reduce the cognitive burden on participants, these choice sets were divided into 4 blocks, with 8 choice sets in each. The participants were randomly assigned to 1 of the 4 blocks: participants only provided their response to the choice sets in the block they were randomized to. In each choice set, participants were asked to choose 1 of the 3 choices (“vaccination plans”), 2 of which were choices to get a COVID-19 vaccine with different attributes, while the third choice was an opt-out option for accepting neither vaccination plan (“no vaccination”). An example of a choice set is shown in Table 2. The questionnaire was validated in the Chinese language. In total, 12 eligible adults were invited to a pilot survey for refinement of the translation in other languages.

**Table 1:** Attributes and levels for the DCE^a^.

Attribute	Levels
Brand	Sinovac BioNTech AstraZeneca
Probability of COVID-19 infection (efficacy)	Reduce 50% infections Reduce 70% infections Reduce 90% infections
Probability of serious adverse event (safety)	1/10,000 1/100,000
Vaccine uptake by people around	Nobody Friends/colleagues Family members
Recommendations from professionals	Recommended by general physicians Recommended by the government expert panel
Venue for vaccination	Community hall Health care facilities Housing estate/workplace
Quarantine arrangement for vaccinated travelers	At least 14-day compulsory quarantine Exempted from the 14-day quarantine

^a^DCE: discrete choice experiment.

**Table 2:** Example of choice sets.

Attribute	Vaccination plan 1	Vaccination plan 2	Do not receive any vaccination
Brand	Sinovac	BioNTech	None
Probability of COVID-19 infection	Reduce 50% infections	Reduce 90% infections	No reduction
Probability of serious adverse event	1/100,000	1/10,000	No serious adverse event
Vaccine uptake by people around	Family members received the vaccine	No known people received the vaccine	N/A^a^
Recommendations from professionals	Government expert advisory panel	General physicians	N/A
Venue for vaccination	Housing estate/workplace	Community hall	N/A
Quarantine arrangement for vaccinated traveler	At least 14-day compulsory quarantine	Exempted from the 14-day quarantine	At least 14-day compulsory quarantine
Which vaccination plan would you choose?	Plan 1: □	Plan 2: □	Neither plan: □

^a^N/A: not applicable.

### Measurement

In addition to measuring the participants’ choice of COVID-19 vaccine via the DCE, the questionnaire also collected data on (1) experience, knowledge, and behaviors during the pandemic, such as whether the participant knew anyone infected with COVID-19, and the perceived susceptibility and severity of COVID-19 infection; (2) experience and perceptions related to vaccination, including previous uptake of influenza vaccination and information sources on COVID-19 vaccination; and (3) demographic and socioeconomic status of the participants, including their ethnicity, years of residence in Hong Kong, income level, education level, and employment status.

### Ethical Considerations

The study was approved by the Survey and Behavioral Research Ethics Committee of the anonymous university (ref. no. SBRE-20-540). At the start of the survey, an electronic informed consent form was provided to the participants with details of the study purpose, data anonymity, and confidentiality. Those who agreed to join signed it electronically before moving to the questionnaire. Later, a supermarket coupon of HK $100 (US $12.74) was sent to each participant through mail. All the responses were anonymized and contained no personal information about any participant. The data were stored online and secured with passwords.

### Statistical Analysis

To find out the preference of all participants for vaccination and its heterogeneity across ethnic groups, a latent-class logit (LCL) model was applied to the entire sample. LCL allocated participants in the survey sample with a similar preference for the vaccination attributes into the same latent class and provided estimates of the preference for each of the classes. The number of latent classes was determined based on the Akaike information criterion (AIC) and Bayesian information criterion (BIC) of the LCL model with the different number of classes. The difference in the preference between the Chinese and non-Chinese participants was explored. Subsequently, the analysis focused on the preference pattern within the non-Chinese migrants due to a substantial difference found between Chinese people and non-Chinese migrants, where the evidence for the latter group was limited.

For migrants, COVID-19 “vaccine refusal” irrespective of vaccine attributes was considered when a participant consistently chose “no vaccination” throughout all 8 choice sets. The vaccine refusal rate across different socialdemographic characteristics and the perception and experience during the COVID-19 pandemic were summarized using cross-tabulation and the chi-square test. To find out the influence of vaccination attributes, a nested logistic model (NLM) was adopted to simulate the choices over the vaccination plans in the DCE survey, which involved 2 decisions, namely (1) whether to accept a COVID-19 vaccine and (2) which COVID-19 vaccine to accept. NLM allowed estimation of the influences of both vaccination attributes and individual-level factors on the vaccine acceptance. The dependent variable was the binary choice (0=“not choose”, 1=“choose”) made for each of the alternatives in the DCE choice sets, and the independent variables were vaccination attributes and individual-level factors. The individual vaccine acceptance probability was estimated based on NLM outcomes [25,26]. In addition, sensitivity analysis was performed using the LCL model among migrant participants to find out whether there was any preference heterogeneity.

## Results

### Sample Characteristics and COVID-19 Vaccine Refusal

In total, 2892 Chinese people and non-Chinese migrants were invited, of which 2392 (82.7%) were eligible. Among them, 434 (18.1%) refused to participate in the survey and 261 (10.9%) did not complete the questionnaire; hence, 2032 (84.9%) valid responses were received.

**Figure 1:**
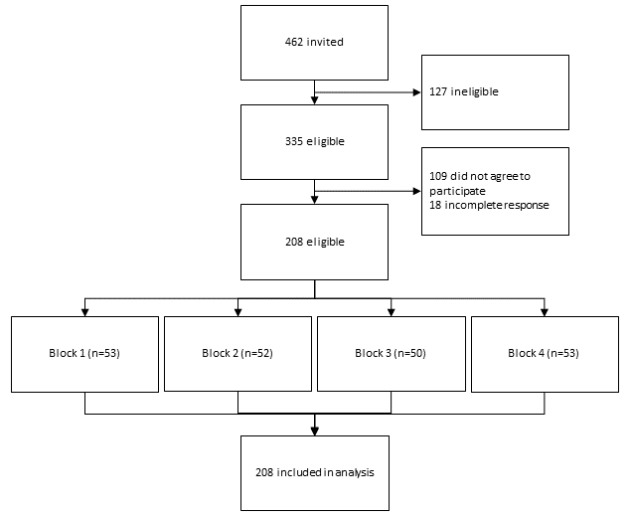
Participant selection flowchart.

Figure 1 shows the participant flow of the migrant group. A total of 462 people received the invitation, and 335 (72.5%) were eligible to the survey. Among them, 109 (32.5%) did not agree to participate and 18 (5.4%) did not complete the questionnaire; therefore, 208 (response rate 62.1%) participants were recruited. The characteristics of the participants can be found in Table 3. Of the 208 participants, 143 (68.8%) were female, and 67 (32.2%) were aged 18-29 years, 103 (49.5%) were aged 30-44 years, and 38 (18.3%) were aged 45 years or more. Most of them (n=180, 86.5%) reported their ethnicity as Asian. In addition, 108 (51.9%) of them attained a bachelor’s degree or above. Almost half of them (n=100, 48.1%) had a full-time job, and 33 (15.9%) were full-time homemakers. Furthermore, 76 (36.5%) of them had more than HK$ 30,000 (US $3821.68) as their monthly household income, which was the approximate median monthly household income in Hong Kong in 2020. Only 19 (9.1%) had chronic conditions. For years of residence, 112 (53.8%) had lived in Hong Kong for 10 years or more, while 31 (14.9%) of them had lived here for 3 years or less.

Regarding COVID-19 vaccine refusal irrespective of vaccination attributes (Table 3), participants with longer local residential years, lower education level, and lower income were more likely to refuse COVID-19 vaccination. With regard to ethnicity, Asian participants were more likely to refuse vaccination than non-Asian participants, although this difference was marginal. Full-time homemakers/housewives, those who had chronic conditions, those who did not know anyone having COVID-19, and those who did not receive influenza vaccines were more likely to refuse vaccination irrespective of the attributes, although the differences were not statistically significant. On the contrary, those with greater perceived susceptibility of COVID-19 infection were less likely to refuse vaccination.

**Table 3:** Sample characteristics and COVID-19 vaccine refusal irrespective of vaccine attributes.

Characteristics			No refusal, n (%)		Refusal irrespective of attributes, n (%)		Migrants (N=208), n (%)		Total participant (N=2032), n (%)
**Age (years), *P*=.26**									
	18-29	56 (83.6)		11 (16.4)		67 (32.2)		832 (40.9)	
	30-44	83 (80.6)		20 (19.4)		103 (49.5)		706 (34.7)	
	45-59	25 (69.4)		11 (30.6)		36 (17.3)		361 (17.8)	
	≥60	1 (50.0)		1 (50.0)		2 (1.0)		133 (6.6)	
**Sex, *P*=.84**									
	Male	51 (78.5)		14 (21.5)		65 (31.2)		769 (37.8)	
	Female	114 (79.7)		29 (20.3)		143 (68.8)		1263 (62.2)	
**Ethnicity^a^, *P*=.06**									
	Asian	139 (77.2)		41 (22.8)		180 (86.5)		2004 (98.6)	
	Non-Asian	26 (92.9)		2 (7.1)		28 (13.5)		28 (1.4)	
**Years of residence in Hong Kong, *P*=.03**									
	≤3 years	28 (90.3)		3 (9.7)		31 (14.9)		N/A^b^	
	4-6 years	28 (93.3)		2 (6.7)		30 (14.4)		N/A	
	7-9 years	27 (79.4)		7 (20.6)		34 (16.3)		N/A	
	≥10 years	81 (72.3)		31 (27.7)		112 (53.8)		N/A	
	Missing	N/A		N/A		1 (0.4)		N/A	
**Education, *P*=.01**									
	Below bachelor’s degree	71 (71.7)		28 (28.3)		99 (47.8)		923 (45.5)	
	Bachelor’s degree or above	93 (86.1)		15 (13.9)		108 (52.2)		1108 (54.6)	
**Employment, *P*=.11**									
	Full-time	81 (81.0)		19 (19.0)		100 (48.1)		1353 (68.6)	
	Part time	28 (80.0)		7 (20.0)		35 (16.8)			
	Unemployed	12 (80.0)		3 (20.0)		15 (7.2)		83 (4.2)	
	Students/interns	23 (92.0)		2 (8.0)		25 (12.0)		271 (13.7)	
	Full-time homemakers/housewives	21 (63.6)		12 (36.4)		33 (15.9)		175 (8.9)	
	Retired	N/A		N/A		N/A		91 (4.6)	
**Monthly household income (HK $/US $)^c^, *P*=.04**									
	<30,000/<3821.68	98 (74.8)		33 (25.2)		131 (63.3)		953 (46.9)	
	≥30,000/≥3821.68	66 (86.8)		10 (13.2)		76 (36.7)		1078 (53.1)	
**Chronic condition, *P*=.07**									
	No	153 (81.0)		36 (19.1)		189 (90.9)		1802 (88.7)	
	Yes	12 (63.2)		7 (36.8)		19 (9.1)		230 (11.3)	
**Know anyone diagnosed with COVID-19, *P*=.07**									
	No	115 (76.2)		36 (23.8)		151 (72.6)		1676 (91.9)	
	Yes	50 (87.7)		7 (12.3)		57 (27.4)		356 (19.5)	
**Perceived “likely/very likely” to be infected, *P*<.001**									
	No	86 (69.9)		37 (30.1)		123 (59.1)		1163 (63.8)	
	Yes	79 (92.9)		6 (7.1)		85 (40.9)		869 (47.6)	
**Perceived “slightly severe/very severe” if get infected COVID-19, *P*=.82**									
	No	80 (80.0)		20 (20.0)		100 (48.1)		978 (53.6)	
	Yes	85 (78.7)		23 (21.3)		108 (51.9)		1054 (57.8)	
**Previous influenza vaccination, *P*=.44**									
	No	138 (78.4)		38 (21.6)		176 (84.6)		1636 (89.7)	
	Yes	27 (84.4)		5 (15.6)		32 (15.4)		396 (21.7)	
**Often received vaccine information from social media, *P*=.60**									
	No	77 (77.8)		22 (22.2)		99 (47.6)		956 (52.4)	
	Yes	88 (80.7)		21 (19.3)		109 (52.4)		1076 (59.0)	
**Often received vaccine information from family/friends, *P*=.06**									
	No	114 (83.2)		23 (16.8)		137 (65.9)		1237 (67.8)	
	Yes	51 (71.8)		20 (28.2)		71 (34.1)		795 (43.6)	
**Often received vaccine information from the workplace, *P*=.33**									
	No	94 (77.1)		28 (23.0)		122 (58.7)		1339 (73.4)	
	Yes	71 (82.6)		15 (17.4)		86 (41.3)		693 (38.0)	
**Often received vaccine information from the government's official source, *P*=.45**									
	No	97 (77.6)		28 (22.4)		125 (60.1)		1292 (70.8)	
	Yes	68 (81.9)		15 (18.1)		83 (39.9)		740 (40.6)	
	Total	165 (79.3)		43 (20.7)		208 (100.0)		1824 (100.0)	

^a^Ethnicity groups included Asians (South Asians, Southeast Asians, and Northeast Asians) and non-Asians (Europeans, Americans, and Africans).

^b^N/A: not applicable.

^c^HK $1=US $0.13.

### Influence of COVID-19 Vaccination Attributes on Vaccine Acceptance Among the Entire Sample

A model with 6 latent classes was applied to test the preference heterogeneity between Chinese people and non-Chinese migrants (Table 4). Using class 6 as a reference, the migrants were much more likely to be assigned to class 1 based on their preference, followed by class 2 and class 4. In class 1, vaccine brand was the most important attribute that affected their choices, where BioNtech was the most preferable brand, followed by vaccine efficacy, venue of vaccination, vaccine uptake by others, and quarantine arrangement for vaccinated travelers. In class 2, vaccine brand, efficacy, safety, and quarantine arrangement for vaccinated travelers shared a similar level of perceived importance based on the respondents’ choices, while vaccination at health care facilities and vaccination at the housing estate/workplace were preferable options as well. In class 4, higher vaccine efficacy was more important than the other attributes that lead to vaccine acceptance. The different preference pattern across classes 1, 2, and 4 implied that preference heterogeneity may be found within the migrant group, which is reported in the following sections. Compared to classes 3, 5, and 6, respondents in classes 1, 2, and 4 were more likely to be affected by the venue of vaccination. In class 3, the participants were efficacy oriented, preferred BioNtech and AstraZeneca, and were more likely to be influenced by general physicians with regard to vaccination decisions. In class 5, the participants preferred Sinovac, were more safety oriented, and presented a lower tendency to refuse vaccination than the other classes. Class 6 had the largest class share, where the participants shared similar preferences with participants in class 3 but were not influenced by recommendations from different health care professionals and vaccine uptake by their family members.

**Table 4:** Preference heterogeneity for COVID-19 vaccination plans in both Chinese people and non-Chinese migrants.

Attribute			Class 1, coefficient^a^ (95% CI)		Class 2, coefficient^a^ (95% CI)		Class 3, coefficient^a^ (95% CI)		Class 4, coefficient^a^ (95% CI)		Class 5, coefficient^a^ (95% CI)		Class 6, coefficient^a^ (95% CI)
**Brand (reference=Sinovac)**													
	BioNtech	3.51^b^ (2.97 to 4.06)		0.49^b^ (0.33 to 0.65)		1.10^b^ (0.87 to 1.32)		1.43^b^ (1.05 to 1.81)		–3.59^b^ (–4.12 to –3.07)		0.57^b^ (0.01 to 1.12)	
	AstraZeneca	–0.29 (–0.73 to 0.16)		0.69^b^ (0.52 to 0.85)		1.29^b^ (1.06 to 1.53)		1.41^b^ (0.94 to 1.89)		–4.04^b^ (–4.58 to –3.50)		0.59^b^ (0.03 to 1.15)	
**Efficacy (reference=50%)**													
	Reduce 70% infections	1.15^b^ (0.83 to 1.48)		–0.03 (–0.16 to 0.10)		0.93^b^ (0.69 to 1.16)		2.10^b^ (1.73 to 2.48)		–0.01 (–0.44 to 0.41)		0.20 (–0.38 to 0.78)	
	Reduce 90% infections	0.56^b^ (0.05 to 1.07)		0.74^b^ (0.58 to 0.91)		2.32^b^ (2.06 to 2.59)		3.43^b^ (2.93 to 3.94)		0.30 (–0.14 to 0.75)		1.12^b^ (0.58 to 1.65)	
**Serious adverse event (reference=1/10,000 people)**													
	1/100,000 people	–0.20 (–0.49 to 0.08)		0.62^b^ (0.51 to 0.72)		0.64^b^ (0.49 to 0.80)		–0.33^b^ (–0.55 to –0.11)		0.33^b^ (0.01 to 0.64)		0.72^b^ (0.26 to 1.18)	
**Vaccine uptake by others (reference=no known people take the vaccine)**													
	Friends/colleagues received	0.58^b^ (0.03 to 1.13)		–0.05 (–0.18 to 0.08)		0.33^b^ (0.14 to 0.52)		1.17^b^ (0.84 to 1.51)		0.64^b^ (0.24 to 1.04)		0.73^b^ (0.19 to 1.28)	
	Family members received	0.63^b^ (0.17 to 1.09)		0.08 (–0.07 to 0.23)		0.42^b^ (0.21 to 0.63)		0.97^b^ (0.59 to 1.34)		0.48^b^ (0.03 to 0.94)		0.41 (–0.21 to 1.03)	
**Recommendations from experts (reference=from general physicians)**													
	From expert advisory panel of the government	–0.13 (–0.58 to 0.31)		0.04 (–0.06 to 0.14)		–0.21^b^ (–0.36 to –0.06)		0.82^b^ (0.53 to 1.11)		0.10 (–0.21 to 0.41)		–0.13 (–0.58 to 0.33)	
**Venue for vaccination (reference=community center)**													
	Health care facilities	–1.00^b^ (–1.51 to –0.50)		0.23^b^ (0.09 to 0.36)		–0.09 (–0.28 to 0.09)		–0.63^b^ (–0.96 to –0.29)		–0.08 (–0.45 to 0.28)		0.05 (–0.47 to 0.57)	
	Housing estate/workplace	0.08 (–0.33 to 0.50)		0.25^b^ (0.12 to 0.39)		–0.04 (–0.22 to 0.15)		–0.40^b^ (–0.69 to –0.10)		0.24 (–0.26 to 0.74)		–0.13 (–0.69 to 0.44)	
**Quarantine arrangement for vaccinated traveler (reference=compulsory quarantine required)**													
	14-day compulsory quarantine can be exempted	–0.54^b^ (–0.98 to –0.10)		0.67^b^ (0.55 to 0.79)		0.44^b^ (0.28 to 0.61)		0.19 (–0.03 to 0.42)		–0.11 (–0.46 to 0.25)		0.80^b^ (0.35 to 1.25)	
**Opt out (reference=not opt out)**													
	Opt out/no vaccination	5.07^b^ (2.35 to 7.80)		1.70^b^ (0.87 to 2.54)		10.07^b^ (8.72 to 11.42)		9.95^b^ (7.43 to 12.48)		–8.24^b^ (–10.27 to –6.21)		10.67^b^ (7.49 to 13.85)	
Class size			10.7% (N/A^c^)		12.2% (N/A)		16.4% (N/A)		21.5% (N/A)		5.8% (N/A)		33.4% (N/A)
**Membership**													
	Age: 30-44 years	1.19^b^ (0.74 to 1.64)		0.31 (0.00 to 0.63)		0.28 (–0.04 to 0.60)		0.58^b^ (0.19 to 0.97)		2.10^b^ (1.21 to 2.99)		Reference	
	Age: 45-60 years	1.57^b^ (1.02 to 2.11)		1.07^b^ (0.68 to 1.46)		–0.04 (–0.58 to 0.50)		1.39^b^ (0.93 to 1.86)		3.24^b^ (2.35 to 4.14)		Reference	
	Age: >60 years	1.80^b^ (1.04 to 2.56)		1.05^b^ (0.43 to 1.66)		–0.15 (–1.03 to 0.74)		1.31^b^ (0.60 to 2.02)		3.78^b^ (2.80 to 4.76)		Reference	
	Bachelor’s degree or higher	0.01 (–0.39 to 0.41)		–0.08 (–0.38 to 0.22)		0.77^b^ (0.43 to 1.11)		0.26 (–0.11 to 0.62)		–0.58^b^ (–1.08 to –0.07)		Reference	
	Monthly income: >HK $30,000/US $3821.68^d^	0.02 (–0.38 to 0.42)		–0.02 (–0.32 to 0.28)		–0.34^b^ (–0.66 to –0.03)		–0.03 (–0.39 to 0.33)		–0.30 (–0.76 to 0.16)		Reference	
	Migrants	1.48^b^ (0.99 to 1.97)		0.86^b^ (0.41 to 1.32)		–0.65 (–1.37 to 0.07)		0.86^b^ (0.34 to 1.38)		–1.55 (–3.32 to 0.23)		Reference	

**^a^**Coefficient of attributes on utility estimated by the latent-class logit (LCL) model. The values of coefficients are weightings of the attribute levels, showing the magnitude of contributions from the attribute levels to the overall utility of the COVID-19 vaccine option. The higher the value is, the greater the contribution the corresponding attribute level makes to the overall utility, and hence the more important and preferable the attribute is.

^b^*P*<.05.

^c^N/A: not applicable.

^d^HK $1=US $0.13.

### Influence of COVID-19 Vaccination Attributes on Vaccine Acceptance Among Migrants

Given that substantial preference heterogeneity was found between Chinese people and non-Chinese migrants, the preference of the migrants was further modeled separately. Among the COVID-19 vaccination attributes, brand, efficacy, safety (probability of serious adverse event), and the quarantine arrangement of vaccinated people could affect vaccine acceptance (Tables 5 and 6). Independent from efficacy and safety characteristics, participants were more likely to accept the BioNtech vaccine compared with Sinovac. The vaccine efficacy also made a substantial difference. A vaccine with 90% and 70% efficacy would increase the likelihood of vaccination by 44% and 21%, respectively. Fewer serious adverse events (1/100,000 vs 1/10,000) could also improve the likelihood of vaccination. Apart from brand, efficacy, and safety, if the quarantine for cross-border travelers can be exempted for those who received the vaccine, the likelihood of vaccine acceptance could increase by around 14%.

**Table 5:** Influence of COVID-19 vaccination attributes on vaccine acceptance in the NLM^a^.

Attribute			Vaccine acceptance, AOR^b^ (95% CI)
**Brand (reference=Sinovac)**			
	BioNtech	1.75^c^ (1.14-2.68)	
	AstraZeneca	1.11 (0.97-1.26)	
**Efficacy (reference=50%)**			
	70%	1.21^c^ (1.03-1.44)	
	90%	1.44^c^ (1.09-1.91)	
**Serious adverse event (reference=1/10,000)**			
	1/100,000	1.12^c^ (1.00-1.24)	
**Vaccine uptake by others (reference=no known people take the vaccine)**			
	Friends/colleagues received	1.11 (0.98-1.25)	
	Family members received	1.12 (0.97-1.28)	
**Recommendations from professionals (reference=general physicians)**			
	Government expert advisory panel	1.08 (0.99-1.17)	
**Venue for vaccination (reference=community center)**			
	Health care facilities	1.00 (0.91-1.10)	
	Housing estate/workplace	0.98 (0.90-1.07)	
**Quarantine arrangement for vaccinated traveler (reference=14-day quarantine)**			
	14-day compulsory quarantine can be exempted	1.14^c^ (1.01-1.30)	

^a^NLM: nested logistic model.

^b^AOR: adjusted odds ratio.

^c^*P*<.05.

**Table 6:** Influence of individual-level factors on vaccine acceptance in the NLM^a^.

Attribute			Vaccine acceptance, AOR^b^ (95% CI)
**Age (years; reference=18-29 years)**			
	30-44	2.42^c^ (1.60-3.64)	
	≥45	2.20^d^ (1.34-3.60)	
Female (reference=male)			1.17 (0.87-1.57)
**Ethnicity (reference=Asian)**			
	Non-Asian	2.28 (0.49-10.64)	
**Years of local residence (reference=less than 3 years)**			
	4-6	0.97 (0.51-1.83)	
	7-9	0.24^c^ (0.13-0.43)	
	≥10	0.41^c^ (0.25-0.67)	
**Education (reference=below bachelor’s degree)**			
	Bachelor’s degree or above	0.76 (0.53-1.09)	
**Employment (reference=full-time)**			
	Part time	1.12 (0.77-1.63)	
	Unemployed	1.08 (0.64-1.81)	
	Students/interns	1.41 (0.81-2.43)	
	Full-time homemakers	0.44^c^ (0.29-0.66)	
**Number of children (reference=0)**			
	1	0.52^d^ (0.33-0.82)	
	2	0.53^d^ (0.33-0.85)	
	≥3	0.36^c^ (0.22-0.61)	
Monthly household income>HK $30,000 (US $3821.68)			1.79^d^ (1.26-2.52)
With any chronic condition			0.61^d^ (0.41-0.91)
Know anyone diagnosed with COVID-19			1.73^d^ (1.25-2.38)
Perceived “likely/very likely” to be infected			3.42^c^ (2.52-4.64)
Perceived “slightly severe/very severe” if get infected COVID-19			0.90 (0.69-1.18)
Previous influenza vaccination			2.15^c^ (1.45-3.19)
Often received vaccine information from social media			1.52^d^ (1.12-2.05)
Often received vaccine information from the workplace			0.42^c^ (0.31-0.57)
Often received vaccine information from family/friends			1.08 (0.80-1.46)
Often received vaccine information from the government's official source			1.27 (0.95-1.70)

^a^NLM: nested logistic model.

^b^AOR: adjusted odds ratio.

^c^*P*<.001.

^d^*P*<.05.

For individual-level factors, participants who had been in Hong Kong for more than 7 years were less likely to accept the COVID-19 vaccine compared with those who had been in Hong Kong for less than 3 years. Full-time homemakers, those with chronic conditions and more children, and those who frequently received vaccine-related information from the workplace were found to be reluctant to accept the vaccine. On the contrary, those with a higher income, those knowing anyone infected with COVID-19, those having greater perceived susceptibility of COVID-19 infection, those who received the influenza vaccine, and those who frequently received information from social media were more likely to accept the vaccine.

Based on the model outcomes, the individual probability of the acceptance of vaccines with different characteristics can be estimated [25,26]. According to the estimation, an Asian migrant aged 18-29 years has a 73.6% chance to accept the BioNtech vaccine with 90% efficacy and a 1/100,000 serious adverse event probability when the vaccine is available in a community center, recommended by the government, and received by family members and when the quarantine exemption is in place. The acceptance probability was estimated to be 48.6% for the Sinovac vaccine with 50% efficacy. A non-Asian aged 18-29 years was estimated to have an 87.1% and a 69.8% chance to accept BioNtech and Sinovac vaccines, respectively. The acceptance probability changes with increasing age. For a migrant aged 30-44 years, the acceptance probabilities were estimated to be 88.9% (BioNtech) and 73.0% (Sinovac) among Asian migrants and 95.1% (BioNtech) and 86.9% (Sinovac) among non-Asian migrants. The estimated probabilities were similar for those aged above 45 years, which were 88.2% (BioNtech) and 71.8% (Sinovac) among Asian migrants and 94.8% (BioNtech) and 86.1% (Sinovac) among non-Asian migrants.

### Sensitivity Analysis Account for Preference Heterogeneity

Substantial preference heterogeneity was found using a 4-class LCL model (Table 7). The participants had a 50.2% probability of belonging to class 1, where they attached greater importance to brand, efficacy, and exemption of quarantine for vaccinated travelers than the other attributes, as found in the NLM model for the entire study sample, and it was considered the reference group for class membership as it had the largest class size. Compared to class 1 (the reference group), participants in class 2 (16.1% probability) were more likely to live in Hong Kong for 7-9 years and they valued safety and the venue for vaccination, in addition to the 3 attributes mentioned in class 1. They were also likely to refuse vaccination (ie, choosing “no vaccination”). Class 2 and class 4 shared similar characteristics, they were likely to have longer local living experience compared to class 1, while class 4 was slightly more likely to comprise full-time homemakers. In class 4 (22.7% probability), the participants did not have clear preferences for these vaccination attributes.

Preferences in class 3 were quite different from those in classes 1 and 2. In class 3 (11.0% probability), participants preferred AstraZeneca over Sinovac and BioNtech and vaccine uptake by family and friends/colleagues as well as recommendations from experts from the government panel could also improve their COVID-19 vaccine acceptance, while exemption of quarantine did not have an impact on it. Participants in class 3 were more likely to be non-Asians and much less likely to be full-time homemakers.

**Table 7:** Sensitivity analysis for preference heterogeneity for COVID-19 vaccination plans.

Attribute			Class 1, coefficient^a^ (95% CI)		Class 2, coefficient^a^ (95% CI)		Class 3, coefficient^a^ (95% CI)		Class 4, coefficient^a^ (95% CI)
**Preference for brand (reference=Sinovac)**									
	BioNtech	1.61^b^ (1.35 to 1.88)		2.13^b^ (1.57 to 2.69)		–0.87 (–1.81 to 0.06)		–0.52 (–3.28 to 2.24)	
	AstraZeneca	0.36^c^ (0.08 to 0.65)		–0.04 (–0.67 to 0.59)		1.21^c^ (0.08 to 2.33)		0.94 (–1.69 to 3.57)	
**Efficacy (reference=50%)**									
	Reduce 70% infections	0.85^b^ (0.57 to 1.12)		0.53^c^ (0.01 to 1.06)		–1.52^c^ (–2.78 to –0.27)		0.12 (–3.75 to 4.00)	
	Reduce 90% infections	1.15^b^ (0.86 to 1.44)		0.62^c^ (0.07 to 1.18)		0.63 (–0.13 to 1.39)		2.67 (–0.26 to 5.60)	
**Serious adverse event (reference=1/10,000 people)**									
	1/100,000 people	0.06 (–0.14 to 0.25)		0.68^c^ (0.26 to 1.09)		1.54^c^ (0.48 to 2.61)		–2.53 (–5.74 to 0.68)	
**Vaccine uptake by others (reference=no known people take the vaccine)**									
	Friends/colleagues received	0.13 (–0.15 to 0.41)		0.45 (–0.07 to 0.98)		1.11^c^ (0.20 to 2.02)		1.55 (–1.85 to 4.94)	
	Family members received	0.22 (–0.09 to 0.53)		0.08 (–0.48 to 0.63)		1.63^c^ (0.33 to 2.92)		2.85 (–1.20 to 6.90)	
**Recommendations from experts (reference=general physicians)**									
	From expert advisory panel of the government	0.19 (–0.01 to 0.39)		–0.39 (–0.83 to 0.04)		1.13^c^ (0.36 to 1.90)		–0.37 (–2.29 to 1.55)	
**Venue for vaccination (reference=community center)**									
	Health care facilities	–0.16 (–0.46 to 0.13)		0.64^c^ (0.12 to 1.17)		0.63 (–0.14 to 1.40)		0.95 (–1.36 to 3.26)	
	Housing estate/workplace	–0.03 (–0.30 to 0.25)		0.09 (–0.45 to 0.62)		0.38 (–0.37 to 1.12)		–0.16 (–3.08 to 2.76)	
**Quarantine arrangement for vaccinated traveler (reference=compulsory quarantine required)**									
	14-day compulsory quarantine can be exempted	0.33^c^ (0.09 to 0.58)		0.73^c^ (0.26 to 1.19)		–0.27 (–1.07 to 0.54)		0.58 (–1.97 to 3.13)	
**Opt out (reference=not opt out)**									
	Opt out/no vaccination	–5.96 (–274.19 to 262.27)		7.00^b^ (4.16 to 9.85)		5.93^c^ (0.67 to 11.19)		13.43 (–4.11 to 30.97)	
Class size			50.2% (N/A^d^)		16.1% (N/A)		11.0% (N/A)		22.7% (N/A)
**Membership**									
	Age: 30-44 years	Reference		–0.93 (–1.92 to 0.06)		–1.23 (–2.46 to 0.00)		–0.35 (–1.27 to 0.56)	
	Age: ≥45 years	Reference		–0.73 (–2.04 to 0.59)		–0.74 (–2.17 to 0.69)		0.15 (–0.97 to 1.26)	
	Non-Asian	Reference		–0.78 (–2.42 to 0.85)		1.20^c^ (0.05 to 2.36)		–0.89 (–2.26 to 0.48)	
	Residential years: 4-6 years	Reference		0.55 (–1.26 to 2.36)		0.79 (–1.26 to 2.84)		0.36 (–1.44 to 2.15)	
	Residential years: 7-9 years	Reference		1.71^c^ (0.08 to 3.35)		0.21 (–2.11 to 2.54)		2.03^c^ (0.48 to 3.57)	
	Residential years: ≥10 years	Reference		1.35 (–0.07 to 2.76)		1.36 (–0.28 to 3.00)		1.69^c^ (0.33 to 3.05)	
	Full-time homemaker	Reference		0.48 (–0.75 to 1.70)		–80.06^b^ (–102.11 to –58.01)		0.96^c^ (0.00 to 1.91)	

**^a^**Coefficient of attributes on utility estimated by the latent-class logit (LCL) model. The values of coefficients are weightings of the attribute levels, showing the magnitude of contributions from the attribute levels to the overall utility of the COVID-19 vaccine option. The higher the value is, the greater the contribution the corresponding attribute level makes to the overall utility, and hence the more important and preferable the attribute is.

^b^*P*<.001.

^c^*P*<.05.

^d^N/A: not applicable.

## Discussion

### Principal Findings

This study investigated both vaccine-related attributes and individual-level factors to fully understand migrants’ COVID-19 vaccine preferences in Hong Kong. It also provided an insight into the heterogeneity of the vaccine preference of the migrant population by dividing them into 4 classes.

### Attributes

Our DCE study found that brand, efficacy, and quarantine arrangement for vaccinated people to be the most relevant vaccine-related attributes for vaccine acceptance among the migrant population in Hong Kong. The heterogeneity analysis complemented these findings, as half of the participants belonging to class 1 made their preferences based on these attributes. In addition, participants in class 3 preferred AstraZeneca over Sinovac and BioNtech. This indicated that migrants are inclined to use the brand as a proxy of unobserved characteristics in decision-making for vaccine acceptance. Previous research conducted on the US general population similarly showed brand and, specifically, the country where the vaccine was developed as predictors of COVID-19 vaccine acceptance [17,27]. With regard to efficacy, our study showed that the higher the efficacy, the higher the willingness to get vaccinated against COVID-19; this is in line with what emerged from studies in the general and ethnic populations in the United States [27] and the United Kingdom [17], where most of these studies have been conducted. Furthermore, specific migrant and ethnic groups may be persuaded if vaccines are proved to reduce the risk of being infected with COVID-19, as shown by research conducted in the United Kingdom [11]. The exemption of quarantine for vaccinated travelers was a predictor of COVID-19 vaccine acceptancy; this result is understandable within the Hong Kong context, where, upon returning, unvaccinated residents undergo self-paid 21-day compulsory quarantine in designated hotels compared to only 7 days for those vaccinated [28]. Other studies did not mention the quarantine arrangement specifically but more generally confirmed the vaccine would be accepted if it enabled social and family life to return to normal [11]. In addition to Sinovac and efficacy, our study found that a lower likelihood of serious adverse events is an attribute for vaccine acceptance, particularly for class 2 and class 3 participants. Similarly, the literature showed that concerns about vaccine safety and side effects are a predictor of vaccine hesitancy in both general and migrant populations in the United Kingdom [6,10,11].

The vaccine uptake by friends, colleagues, and family members; information from family and friends; and recommendations from the government and health care professionals were insignificant for the entire population sample but significantly affected only class 3 participants’ vaccine acceptance. These participants were mostly non-Asian. The existing literature suggests that such recommendations may be welcomed only in the presence of trust toward the government and the health care system [5,6]. In addition, American Hispanics in the United States, for instance, rely on their community network to make decisions about COVID-19 vaccines [29]. Similarly, migrants in the United Kingdom tend to look for information from peers, especially when the government and health care system are not trusted [5]. To promote COVID-19 vaccinations and improve their health care access and quality, efforts should be made to establish more trustworthy relationships between the local government and the health care system with all the migrant populations in Hong Kong.

In this study, based on vaccine attributes, we did not find significant preference differences between the migrant population and the general population. Instead, there was a substantial heterogeneity representing diversity in the preferences of the non-Chinese migrant population in Hong Kong. This implied that while designing COVID-19 vaccination promotion programs for migrants, this diversity should be considered and more targeted strategies should be used for different migrant subgroups. The venue of vaccination was the only significant factor for vaccine acceptance among migrants when compared to the general population, which is consistent with the literature that suggests an inconvenient location would influence hesitancy [30] and minimal travel or comfortable places would reduce hesitancy among undocumented migrants, asylum seekers, and refugees [6]. However, in the sensitivity analysis for preference heterogeneity among the migrant population, only a small proportion of participants in class 2 of our study had an attached preference with the venue of vaccination. These participants also shared characteristics with class 4 participants who did not have a clear preference for vaccine attributes. This means the choices made by these people were quite random, so they were probably overwhelmed by the choice tasks.

### Individual-Level Factors

Our results also showed that irrespective of the vaccination attributes, longer residential years, lower education and income, and less perceived COVID-19 infection susceptibility were associated with increased COVID-19 vaccine refusal. These individual-level factors were also related to decreased COVID-19 vaccine acceptance among those whose preferences were linked to vaccine attributes. Participants with more than 7 years of migration history in Hong Kong were least likely to get vaccinated compared to those with a more recent migration history. This aligns with much of the literature on migrant health, where a longer history of migration and host country language proficiency are associated with acculturation in the host society and health care use similar to nationals [12]; vaccine hesitancy among Hong Kong local residents is in fact similarly widespread [31]. Therefore, effective policies and strategies that can boost vaccination acceptance among the broader public will aid in achieving high vaccination uptake among migrants as well. Previous research has also identified the educational level as a predictor of vaccine acceptance [9,11]. In line with most research, a higher income was also associated with higher vaccine acceptance, while a lower income was associated with vaccine resistance [10,27]. Similar to what emerged from the previous literature, knowing someone diagnosed with COVID-19 was a factor for vaccine acceptance [17] and so was high risk perception; conversely, low risk perception was associated with higher hesitancy [30].

In addition, our study also identified other individual-level factors that predict COVID-19 vaccine acceptance among individuals who preferred different vaccine programs. A previous influenza vaccination experience was relevant for COVID-19 vaccine acceptance; this is in line with studies conducted in migrant and general populations in other settings [8,10,17].

Our study also indicated that those participants who often received information from social media were more likely to accept COVID-19 vaccination. Regarding the role of this source of information, the literature is mixed, with varying degrees of results; some studies suggest social media is used to solve confusion from contradicting information [5], while other research suggests it is a source of misinformation among migrants [4,6]. Our study also found that those who received information from the workplace were less likely to get vaccinated. This might be because, on the one hand, these people are employed and healthy and therefore have low risk perception, leading to low vaccine acceptance. On the other hand, working people are exposed to different information sources, including local ones; discussions with their colleagues may also increase their vaccination hesitancy, as there is a decreasing trend in the willingness toward COVID-19 vaccination among general working people in Hong Kong [32]. The promotion of vaccine-related information through this source should be encouraged.

Older age was identified as a factor of vaccine acceptance; specifically, participants aged 30-44 years would be the most likely to get vaccinated, while the youngest cohort (18-29 years old) would be the least likely to join the vaccination campaign. A study in the United Kingdom confirmed the young group (16-24 years old) to be the most hesitant [11]; this hesitancy may be motivated by low risk perception compared to other age groups [30]. COVID-19 vaccination promotion campaigns should target young age group specifically among the migrant population in Hong Kong.

In line with the international literature, the Hong Kong Centre for Health Protection encourages individuals with chronic conditions to get vaccinated against COVID-19, since they are at a higher risk of morbidity and mortality due to COVID-19 infection [33]; however, our study highlighted that people with chronic diseases are less likely to accept vaccines. This result suggests the need to plan COVID-19 vaccination promotion campaigns targeting this high-risk group specifically.

Being a homemaker and having children were indicators of COVID-19 vaccine hesitancy; in the United Kingdom, those living with children are similarly less likely to accept the vaccine [10]. This might be because migrants have a lack of family and social support and, being parents, they are main carers of their children. During COVID-19, the care burden of parents, particularly homemakers and mothers, has increased due to lockdown, school closure, and home schooling. They have busy routines and no time for making appointments and going for vaccination. Moreover, there might be concerns about who will take care of their children in case they have vaccine-related side effects. So, along with providing information about vaccine safety, an outreach approach can be considered for this particular migrant subgroup. Qualitative research is needed to further address the reasons behind this attitude.

In this study, gender was not a predictor of COVID-19 vaccine hesitancy; this is in contrast with previous research in other settings that identified women as being more hesitant compared to men both in the general and in the migrant population [8,10,11,17].

### Limitations

Our study had some limitations that should be clarified when interpreting the results. First, the preference of respondents for COVID-19 vaccination is subject to change with time and could be different as the vaccination campaign progresses. It may also have been affected by reported adverse events following immunization for COVID-19, which were not captured in this survey. Second, although we incorporated “Asian”/“non-Asian” as a variable for ethnicity in the analysis (Tables 5 and 6), there were no significant difference in vaccine acceptance between these 2 groups, and due to the limited sample size of ethnicity subgroups and statistical power, we could not further subdivide the ethnicity in our analysis. Instead, the LCL model was used to identify the preference heterogeneity (Table 7), where non-Asian migrant respondents tended to attach more importance to the vaccination of family and friends and preferred AstraZeneca to the other 2 vaccine brands compared to Asian migrants. However, the ethnicity of the respondents could not be further divided in the analysis due to the limited subgroup sample size. Third, although the survey was piloted and refined prior to the formal investigation, there were around 20% respondents (class 4 in the LCL model) who may have been overwhelmed by the cognitive burden of the DCE: their COVID-19 vaccination choice appears to be random. By using the LCL model, we were able to separate them from other groups of respondents and avoid affecting the results.

### Conclusion

In summary, this study provided insight into how different COVID-19 vaccine attributes, preferences, and individual-level characteristics of the migrant population could influence their COVID-19 vaccine acceptance. Although vaccine brand, efficacy, safety, and quarantine exemption were the most preferred attributes for the majority of the migrants, we reiterate that there is COVID-19 vaccination preference heterogeneity among migrants and that more targeted and tailored approaches are needed to promote vaccine acceptance for different subgroups of the migrant population in Hong Kong. The increased willingness toward COVID-19 vaccination in the general population will reduce vaccine hesitancy among migrants with long years of living experience in Hong Kong. Vaccination promotional programs for low-education and low-income migrant groups should be designed in different languages. The dissemination of COVID-19 vaccine information, particularly about efficacy and safety, through social media platforms can be continued, and information dissemination at the workplace for the working migrant population should also be considered. Detailed and tailored educational programs should be provided to people with chronic diseases to promote COVID-19 vaccination in this high-risk group. An outreach approach for people with children, particularly homemakers and mothers, can be used. Further follow-up studies, including qualitative interviews, can be conducted with these participants to explore in-depth the reasons behind their COVID-19 vaccination preferences and refusal.

## Abbreviations

AOR: adjusted odds ratio
DCE: discrete choice experiment
LCL: latent-class logit
NLM: nested logistic model

## Data Availability

All the data used in this study are available upon request from the corresponding author.
